# New insights into the mode of action of the lantibiotic salivaricin B

**DOI:** 10.1038/srep31749

**Published:** 2016-08-16

**Authors:** Abdelahhad Barbour, John Tagg, Osama K. Abou-Zied, Koshy Philip

**Affiliations:** 1 Division of Microbiology, Institute of Biological Sciences, Faculty of Science, University of Malaya, Kuala lumpur, Malaysia; 2 Department of Microbiology and Immunology, University of Otago, P.O. Box 56, Dunedin 9054, New Zealand; 3 Department of Chemistry, Faculty of Science, Sultan Qaboos University, P.O. Box 36, Postal Code 123, Muscat, Sultanate of Oman

## Abstract

Salivaricin B is a 25 amino acid polycyclic peptide belonging to the type AII lantibiotics and first shown to be produced by *Streptococcus salivarius*. In this study we describe the bactericidal mode of action of salivaricin B against susceptible Gram-positive bacteria. The killing action of salivaricin B required micro-molar concentrations of lantibiotic whereas the prototype lantibiotic nisin A was shown to be potent at nano-molar levels. Unlike nisin A, salivaricin B did not induce pore formation or dissipate the membrane potential in susceptible cells. This was established by measuring the fluorescence of the tryptophan residue at position 17 when salivaricin B interacted with bacterial membrane vesicles. The absence of a fluorescence blue shift indicates a failure of salivaricin B to penetrate the membranes. On the other hand, salivaricin B interfered with cell wall biosynthesis, as shown by the accumulation of the final soluble cell wall precursor UDP-MurNAc-pentapeptide which is the backbone of the bacterial peptidoglycan. Transmission electron microscopy of salivaricin B-treated cells showed a reduction in cell wall thickness together with signs of aberrant septum formation in the absence of visible changes to cytoplasmic membrane integrity.

Lantibiotics are ribosomally-synthesized antimicrobial peptides containing intramolecular ring structures introduced through the thioether-containing lanthionine (Lan) and/or methyllanthionine (MeLan) residues formed by post-translation modification^1^. Although most of the currently-described lantibiotics are produced by Gram-positive bacteria^2^ certain isolates of *Streptomyces* have also been shown to produce lantibiotics or lantibiotic-like peptides^3^,^4^. Lantibiotics are widely-considered to assist the survival of the host bacteria in their favoured ecosystem by suppressing the growth of competitor bacteria in that particular ecological niche.

The most well-known lantibiotic is nisin, which was first described in 1928^5^ and then subsequently widely used in the dairy industry as an effective and safe preservative^6^. Lantibiotics from gram positive bacteria are classified into two major groups based on their modes of action and structural variations^7^. Nisin, epidermin and Pep5 are members of the type A lantibiotic group and they act mainly by forming pores in the cytoplasmic membrane of the targeted bacterial cells^8^. On the other hand, type B lantibiotics such as mersacidin form complexes with their membrane bound substrates and inhibit peptidoglycan synthesis^9^,^10^,^11^,^12^,^13^. While type AI lantibiotics (the nisin group) are elongated and flexible, type AII (the lacticin 481 group) display an unbridged N-terminal extremity and a globular C-terminal part. Type AIII lantibiotics consists of lactosin S and the two-component system lantibiotics^14^,^15^,^16^.

Salivaricin B is a type AII lantibiotic produced by *Streptococcus salivarius* strain K12 and having a ring topology similar to that of the *Lactococcus lactis* lantibiotic, lacticin 481^16^,^17^,^18^ (Fig. 1).

*S. salivarius* is a commonly-occurring member of the human oral microbiota, typically colonizing the mouth and upper respiratory tract within a few hours of birth. Some *S. salivarius* are equipped to compete with predominant bacterial pathogens involved in upper respiratory tract infections due to their production of various lantibiotics, which include salivaricin A, salivaricin B, salivaricin G32 and salivaricin 9^18^,^19^,^20^,^21^,^22^,^23^,^24^. Salivaricin B is particularly potent, with a broad inhibitory spectrum that includes all 9 standard indicator strains used in the production (P-) typing method that was developed specifically for the categorization of bacteriocin-producing streptococci^18^,^25^.

One important characteristic of the members of the lacticin 481 group is that they contain a mersacidin-like lipid II binding motif and in this regard salivaricin B is no exception^16^,^18^,^26^. Although salivaricin B and lacticin 481 are classified as class AII lantibiotics they also contain an important membrane binding motif found in class B lantibiotics, which makes it interesting to study the mechanism of action of these lantibiotics and to determine whether they follow the typical pore formation activity of class A lantibiotics or interfere with cell wall biosynthesis like class B lantibiotics^7^.

In the present study, molecular probes were used to investigate whether salivaricin B disrupts bacterial cell membrane integrity or dissipates the membrane potential of targeted cells. Spectrofluorometric analysis was also carried out to determine whether the tryptophan residue of salivaricin B plays any role in the peptide-membrane interaction. It is concluded that salivaricin B interferes with cell wall biosynthesis by deregulating the cell envelope and interfering with septum formation.

## Results

### Salivaricin B production, purification and molecular weight determination

*S. salivarius* strain K12 (producer of salivaricins A2 and B) was first tested by the deferred antagonism method to evaluate its *in vitro* inhibitory activity before scaling up lantibiotic production. One μl of an 18 h culture of strain K12 grown in PTNYSMES medium^24^ was spotted on BaCa medium and then allowed to grow for 18 h before being overlaid with the indicator strain. Lantibiotic production by strain K12 was displayed as a zone of inhibition surrounding the producer cell culture, indicating susceptibility of the tested indicator strains (*Micrococcus luteus* ATCC10240, *Streptococcus pyogen*es ATCC12344 and *Corynebacterium spp* GH17) (Fig. 2a). *Streptococcus mutans* GEJ11 was not sensitive to K12 lantibiotics in this assay. Freeze thaw extraction of K12 cultures grown on M17-agarose, followed by hydrophobic interaction chromatography yielded crude lantibiotic preparations containing both salivaricin A2 and salivaricin B. High performance liquid chromatography on a C18 semi-preparative column helped to separate the two lantibiotics at retention times of 50 minutes for salivaricin A2 and 55 minutes for salivaricin B (Fig. 2b). High resolution MALDI-TOF (MS) analysis confirmed the predicted molecular weights. Salivaricin B mass spectrum showed an exact mass of 2732.3867 Da and an average mass of 2733.3899 Da. Salivaricin A2 showed an exact mass of 2366.1946 Da and an average mass of 2367.1975 Da. Both lantibiotics can be seen as single peaks resolution as shown in the supplementary file (Figure S1).

### Minimal inhibitory concentration (MIC), IC50 and time killing assay

Agar well diffusion assays were initially performed to obtain preliminary qualitative data concerning the relative susceptibility of various Gram-positive bacteria to salivaricin B and nisin A. Bacterial strains showing susceptibility towards both lantibiotics in this assay were then subjected to growth inhibition assays in liquid media from which quantitative MIC values were determined. Both salivaricin B and nisin A failed to inhibit Gram-negative bacteria. The diverse specific concentrations of salivaricin B and nisin A required to inhibit individual strains of Gram-positive bacteria indicated that the activity of both lantibiotics is strain-dependent. *Lactococcus lactis* HP has been shown previously to be highly susceptible to nisin A and various other lantibiotics. In this study, nisin A was shown to have a very low nano-molar MIC for this strain (MIC = 39 nM, IC50 = 17.7 ± 0.9) when compared with the relatively modest potency of salivaricin B (MIC = 1080 nM, IC50 = 407 ± 34). On the other hand, the nisin A producer (*L. lactis* ATCC11454) was more sensitive to salivaricin B than to nisin A. Our data showed that salivaricin B is bactericidal for most streptococci in a range of 2160–4320 nM. However, some streptococci such as *S. mutans* exhibited resistance to ≥8 μM of salivaricin B. The list of MIC values obtained in this study can be seen in Table 1. Salivaricin B was bactericidal for both *S. pyogenes* and *M. luteus*. After 30 minutes of salivaricin B exposure, more than 40% of *S. pyogenes* cells were killed indicating a rapid killing activity. Moreover, salivaricin B (10X MIC) killed more than 90% of *S. pyogenes* in less than 3 hours. Similar activity was detected against *M. luteus* but with even stronger potency (Fig. 3). No significant lysis occurred when either *S. pyogenes* or *M. luteus* were treated with 10X MIC levels of salivaricin B.

### Microplate growth inhibition assays

Treating susceptible *S. pyogenes* cells with different concentrations of salivaricin B revealed that the bactericidal effect is concentration-dependent. Cells treated with 2.5 μM salivaricin B failed to propagate, whereas 90% growth inhibition was obtained in cells treated with 2 μM salivaricin B and 50% of *S. pyogenes* cell growth was inhibited by 1.0 μM salivaricin B. Growth was not affected significantly in cells treated with 0.5 μM salivaricin B (Fig. 4a). This assay was also carried out against *M. luteus* cells which were shown to be more susceptible to salivaricin B. *M. luteus* was grown in MHB at 37 °C and monitored for 10 hours, which was the required incubation time for *M. luteus* cells to reach OD_600_ = 0.4 in the microplate growth inhibition assay. Complete inhibition of *M. luteus* growth was achieved with only 0.5 μM of salivaricin B and 0.125 μM affected 70% killing Fig. 4b).

### Assessment of pore formation using SYTOX Green-labeled cells

SYTOX Green probe is a high affinity DNA stain which is impermeable to cells with intact membranes. However, when pores are formed in the targeted membranes by a pore forming agent, the probe enters the cells and interacts with the genomic DNA to generate increased fluorescence intensity. In this study, we probed potential membrane permeabilization by salivaricin B in *S. pyogenes* ATCC1234 using SYTOX Green. Nisin A when used at 5-fold MIC gave an immediate large increase in cell-associated fluorescence intensity (FI) consistent with loss of membrane integrity and pore formation. In contrast, salivaricin B was unable to increase FI above control levels at concentrations up to 10-fold MIC value. It was noticed that the FI of nisin-treated cells increased gradually with time indicating disruption of membrane integrity of more cells during an extended incubation period whereas no significant increment of the FI of salivaricin B-treated cells was observed even after 30 minutes of incubation (Fig. 5). Similarly, unlike nisin A, salivaricin B (10X MIC) did not induce pore formation in *M. luteus* cell membranes in this study even with extended incubation (30 minutes). The results provide clear evidence that salivaricin B is not able to induce pore formation in sensitive cells.

### Dissipation of membrane potential

The ability of salivaricin B to dissipate the membrane potential of the targeted bacterial cells was investigated in this study using DiOC2(3) (3,3-diethyloxacarbocyanine iodide). This molecular probe exhibits green fluorescence in all bacterial cells, but the fluorescence shifts towards red emission as the dye molecules self-associate at the higher cytosolic concentrations caused by higher membrane potentials. Analysis of DiOC2(3)-labeled *M. luteus* ATCC10240 cells was carried out using scatter plots of green versus red fluorescence. Previous reports of the depolarization ability of nisin A showed similar results to the present flow cytometric study in that *M. luteus* cells were significantly depolarized by nisin A. Figure 6 shows both intact and depolarized population due to nisin pore formation activity. In the case of exposure to salivaricin B (10-fold MIC) however, no changes in the population location could be distinguished from intact cells indicating no membrane potential dissipation occurred. These findings provide strong evidence that salivaricin B is not able to damage the membrane integrity of salivaricin B-susceptible bacterial cells.

### Tryptophan fluorescence

Interaction of the salivaricin B peptide with bacterial membranes was investigated by measuring the fluorescence change of tryptophan. The fluorescence peak position of tryptophan reflects the medium polarity and can be used to predict the local environment around the tryptophan residue in a biological system^27^,^28^. The lowest energy of the fluorescence peak maximum is at ~355 nm and this is usually taken as an indication of the exposure of tryptophan to aqueous solution.

Figure 7 shows the fluorescence spectra of the aqueous-buffer solution of salivaricin B, *B. cereus* membrane, *E. coli* membrane, and the mixtures of salivaricin B with the membranes. The peak location of the salivaricin B peptide is at ~355 nm, while the spectral locations of the two membranes are at 348 and 336 nm for *B. cereus* and *E. coli* membranes, respectively. The latter values indicate the presence of partially buried tryptophan(s) in the two membranes, whereas in salivaricin B the peak at 355 nm is indicative of exposed tryptophan(s) to buffer. Upon mixing the peptide with the membranes, there is a slight increase in intensity, relative to that of the membranes alone, which indicates the presence of an interaction between the peptide and the two target membranes. On the other hand, the spectral location of the fluorescence peak in each mixture remains the same, within the measurements of uncertainty, as that of the corresponding membrane alone.

It is always difficult to correlate the change in fluorescence to the actual mode of peptide-membrane attack when more than one tryptophan residue is present^29^. Nevertheless, comparing the spectral change in the absence and presence of the peptide indicates that the mode of binding between the peptide and the membrane is not vertical (penetration) and more likely to be parallel. If the attack is a perpendicular penetration, a blue shift is usually expected in the fluorescence peak of tryptophan, compared to that of the peptide alone as we recently observed in other systems^30^.

### Accumulation of the final soluble cell wall precursor UDP-MurNAc-pentapeptide

In order to establish whether salivaricin B interferes with peptidoglycan biosynthesis, the cytoplasmic level of the cell wall precursor UDP-MurNAc-pentapeptide in *M. luteus*-treated cells was determined. Usually, accumulation of this cell wall precursor is induced by antibiotics such as vancomycin, which inhibit the late membrane-bond steps of cell wall biosynthesis. In this study, salivaricin B induced accumulation of cell wall precursor when compared to non-treated cells, as revealed by reverse phase high performance liquid chromatography (RP-HPLC). Extracts of vancomycin-treated cells served as a positive control for this test. Identification of UDP-MurNAc-pentapeptide was carried out using mass spectroscopy (Figure S2). This study showed that like vancomycin, salivaricin B induces the accumulation of the final soluble cell wall precursor UDP-MurNAc-pentapeptide and ultimately interferes with peptidoglycan biosynthesis in susceptible cells as shown in Fig. 8. It was noticed that in Fig. 8c additional peaks (8–10 min) were present when the salivaricin B-treated cells were extracted with boiled water. This could be additional cytoplasmic materials accumulated when cells were exposed to salivaricin B. It was also noticed that when salivaricin B-treated *M. luteus* cells were extracted with water at boiling temperature after which there was slight change in the biomass color from yellow (typical of *M. luteus* cells) to light brown. Possibly excess peptide became embedded in the cell envelopes of those cells that had been exposed to the boiling water. As a result, the additional peaks (at 8–10 minutes) in Fig. 8C could be denatured peptides’ fragments extracted with the cells.

### Ultrastructural modification and inhibition of cell wall biosynthesis

After 30 minutes of salivaricin B exposure, most of the *S. pyogenes* ATCC12344 cells did not show significant damage or ultrastructural changes when compared to untreated control cells except for some changes in the polar sides of the cells which developed a sharper shape when compared to the smooth spherical shape of the control cells. However, less than 5% of the cells showed additional and aberrant division septa after 120 minutes of salivaricin B treatment. No sign of membrane damage or detachment of the cell wall from the cytoplasmic membrane was observed even after 120 minutes of salivaricin B treatment. However, some *S. pyogenes* cells showed thinning cell wall and partial lysis (Fig. 9). We used another indicator strain, *M. luteus* ATCC10240, which is more susceptible to salivaricin B than *S. pyogenes. M. luteus* cells treated with salivaricin B showed aberrant division, where the cells were divided into many sections and expanded without losing membrane integrity, although the cell walls were drastically reduced in thickness. Compared with nisin A-treated cells, salivaricin B-treated *M. luteus* cells did not show any sign of membrane damage. On the contrary, immediately after the addition of nisin A, *M. luteus* cells started to get depolarized membranes due to the pore formation mechanism exhibited by nisin A as its principal mode of action^31^,^32^ (Fig. 10). It was very clear that the inner cytoplasmic material of nisin A-treated cells oozed out of the cells when observed by TEM.

## Discussion

**Figure 1:**
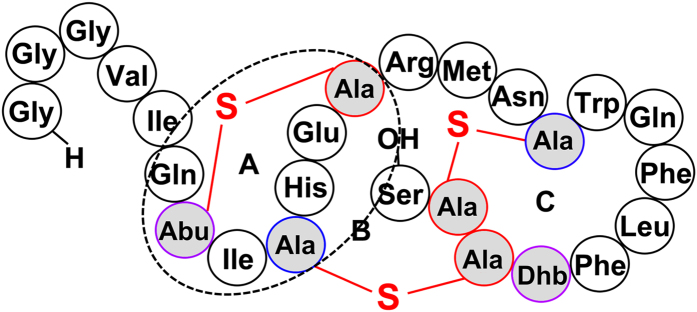
A proposed structure of salivaricin B lantibiotic based on the resolved structure of lacticin 481. S, thioether sulfur. Dehydrated residues and residues linked by lanthionine rings are highlighted. Mersaciin lipid II-binding motif is indicated in black broken lined circle. [Ala]-S-[Ala]: lanthionine, [Abu]-S-[Ala]: β-methyllanthionine, [Dhb]: dehydrobutyrine.

**Figure 2:**
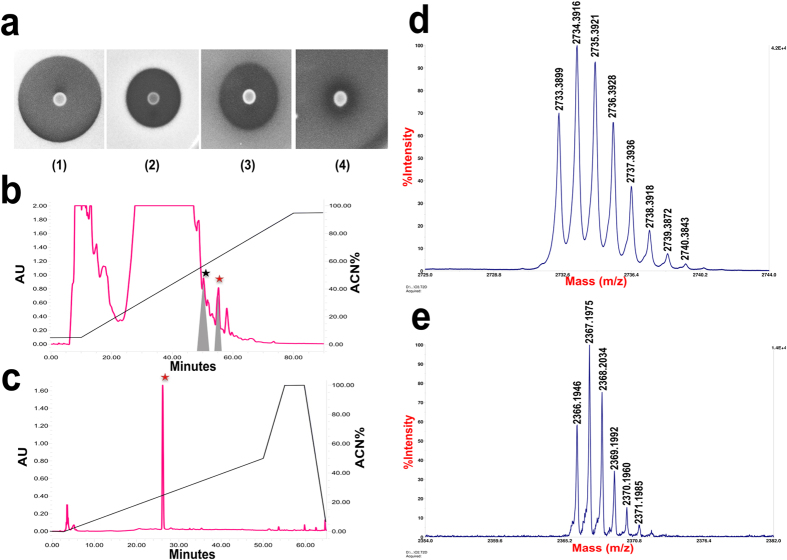
Production and purification of salivaricin B. (**a**) Deferred antagonism assay on blood agar. The salivaricin B producer strain (K12) was spotted first at the center of blood agar, after growth it was overlaid with the indicator strains *Corynebacterium spp*1, *Micrococcus luteus* 2, *Streptococcus pyogenes* 3 and *Streptococcus mutans* 4. (**b**) Semi-preparative separation of salivaricin A2 (black star) and salivaricin B (red star). Inhibitory activity is shaded in the two peaks. (**c**) Purity check of salivaricin B using Aeris PEPTIDE column. (**d,e**) MALDI-TOF (MS) analysis of salivaricin B and salivaricin A2 respectively.

**Figure 3:**
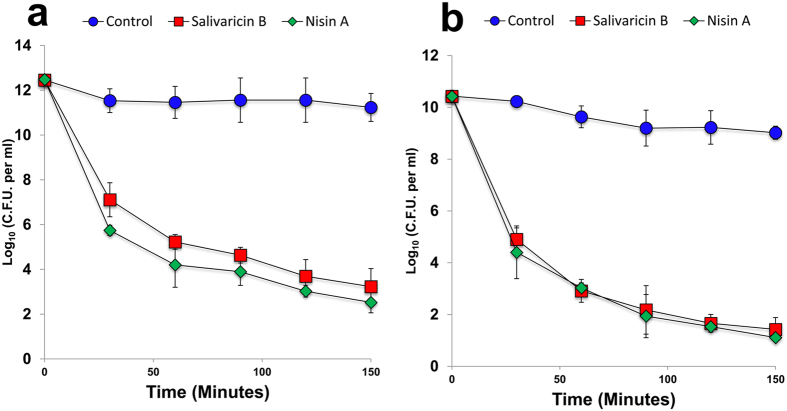
Time killing assay of salivaricin B against *S. pyogenes* (**a**) and *M. luteus* (**b**).

**Figure 4:**
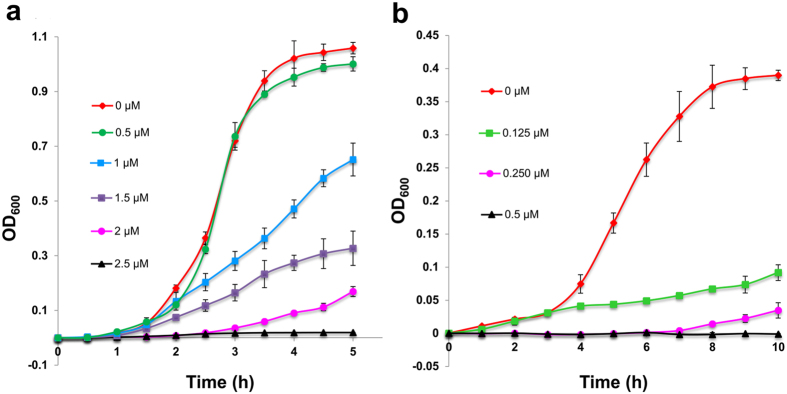
Inhibitory activity of salivaricin B in microplate growth inhibition assay against *S. pyogenes* ATCC1234 (**a**) and *M. luteus* ATCC (**b**). Where error bar is invisible, the error is smaller than the symbol used.

**Figure 5:**
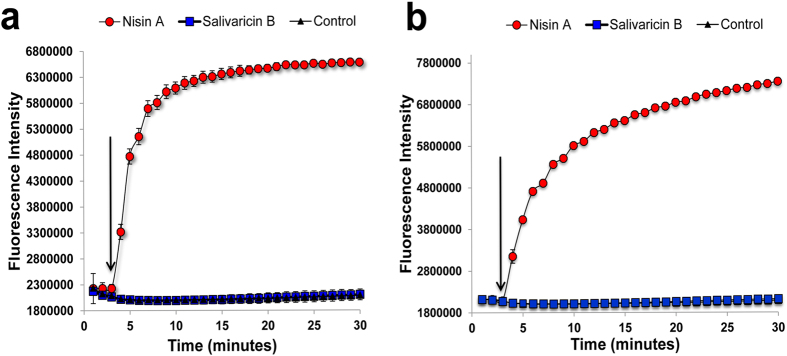
Pore formation assay. Unlike nisin A, salivaricin B did not induce pore formation as no detectable SYTOX green fluorescence could be observed for cells exposed to salivaricin B in both *M. luteus* (**a**) and *S. pyogenes* (**b**). Arrows indicate the time of lantibiotic addition.

**Figure 6:**
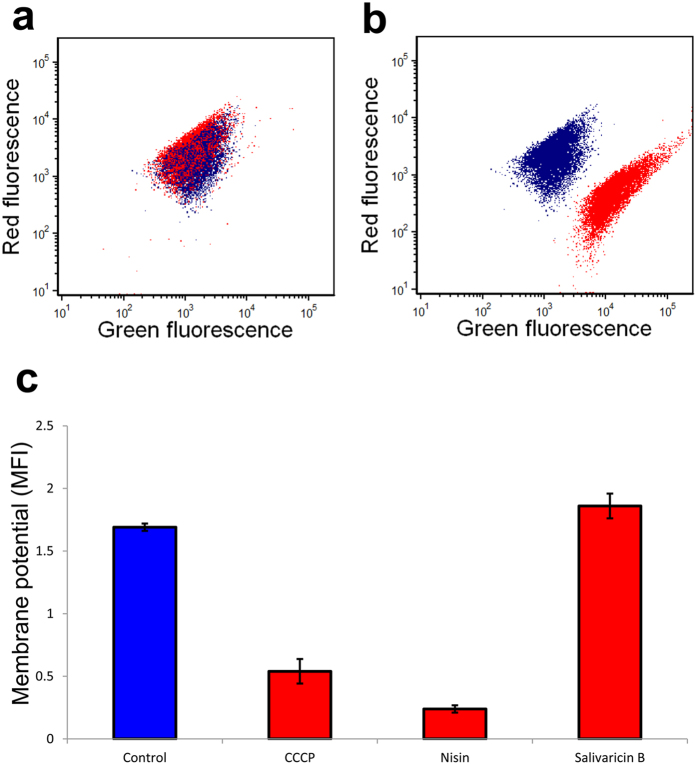
Cytometric profiles of *M. luteus* cells labeled with DiOC2(3). Blue dots represent healthy cells with no lantibiotic added. Red dots represent lantibiotic-treated cells. Comparison between healthy and salivaricin B treated cells (**a**). Comparison between healthy and nisin treated cells (**b**). Geometric means of fluorescence intensity of DiOC2(3) labeled *M. luteus* cells treated with different antimicrobials (**c**).

**Figure 7:**
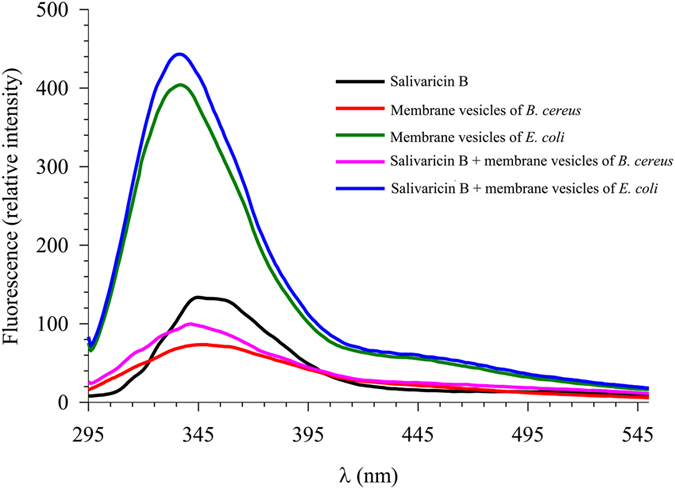
Fluorescence spectra of salivaricin B in the absence and presence of different bacterial membrane vesicles. The concentration of membrane vesicles and peptides was 0.05 mM in 10 mM phosphate buffer pH 7.4.

**Figure 8:**
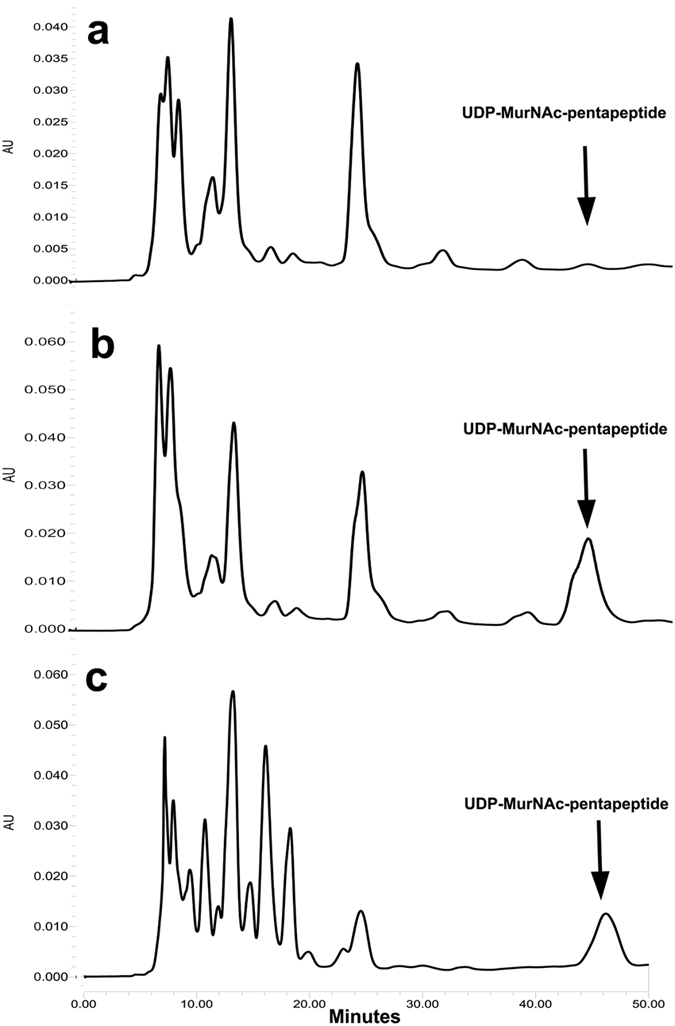
Intracellular accumulation of the final soluble cell wall precursor UDP-MurNAc-pentapeptide in *M. luteus* ATCC10240 exposed to salivaricin B. Cells were treated with vancomycin (10 × MIC) (**b**) or salivaricin B (**c**), incubated for 60 min, and subsequently extracted with boiling water. The cytoplasmic pool of UDP-linked cell wall precursors was analysed by RP-HPLC. Bacterial culture with no antibiotic added served as a control (**a**).

**Figure 9:**
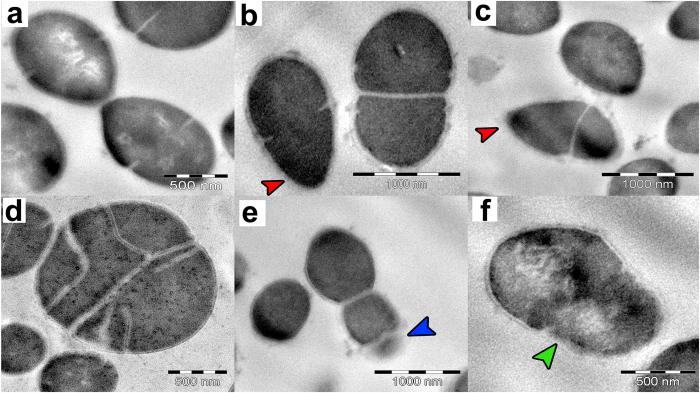
Ultrastructural effect of salivaricin B towards *S. pyogenes* cells. (**a**) Control (untreated), (**b–d**) Salivaricin B treated cells (30 minutes), (**e**) salivaricin B treated cells (120 minutes), (**f**) Nisin A treated cells. Red arrows indicate change in the typical spherical shape of *S. pyogenes* cells. Blue arrow indicates partial lysis of the cell. Green arrow indicates depolarized membrane due to nisin activity.

**Figure 10:**
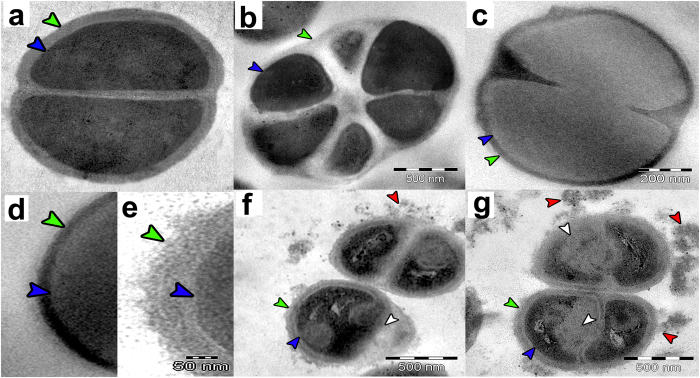
Ultrastructural effect of salivaricin B towards *M. luteus* cells. (**a**) Control (untreated), (**b,c**) Salivaricin B treated cells, (**d**) Cell wall of salivaricin B treated cell, (**e**) Cell wall of untreated cell, (**f,g**) Nisin A treated cells. Green arrows indicate cell wall. Blue arrows indicate cell membrane. Red arrows indicate inner cytoplasmic materials oozed out of the cell. White arrows indicate membrane disruption.

Generally, lantibiotics exert their inhibitory activity against the targeted bacterial cells by disturbing the integrity of the cytoplasmic membrane through pore formation^33^,^34^,^35^. This mechanism is usually facilitated by using the peptidoglycan precursor lipid II as a docking site^36^,^37^. However, the mechanisms of action of many lantibiotics belonging to different subclasses are largely unexplored. Salivaricin B, a class AII lantibiotic produced by *S. salivarius,* was shown in this study to exhibit a different mode of action than the typical pore formation. It is usually considered that lantibiotics having a bactericidal mode of action will interfere with the cytoplasmic membrane integrity of susceptible cells. However, lacticin 481 was shown previously to interfere with peptidoglycan biosynthesis by inhibiting transglycosylation without forming pores in the membranes of susceptible bacteria^26^. Like lacticin 481, many other lantibiotics belonging to the same group e.g. nukacin ISK-1, mutacin II and streptococcin SA-FF22 contain a lipid II binding motif (TXS/TXD/EC) present in mersacidin (class B lantibiotic) where X can be any residue. Salivaricin B also has this motif at its ring A (Fig. 1) which makes lipid II its likely target. Previously it was reported that nukacin ISK-1 has a bacteriostatic mode of action towards *B. subtilis* JCM 1465^T^ cells without causing pore formation or membrane potential dissipation^38^. However, it was found that nukacin ISK-1 can also exhibit a bactericidal mode of action with pore formation ability when tested against *M. luteus* DSM 1790 and *Staphylococcus simulans* 22^39^. Mutacin II was shown to possess a bactericidal mode of action by partially depolarizing the transmembrane electrical potential (∆Ψ) which then recovered shortly after *S. sanguis* Ny101 cells were treated with mutacin II^40^. It was shown previously that streptococcin SA-FF22 can form relatively unstable, short-lived pores of diameter approximately 0.5–0.6 nm and dissipate the membrane potential with 100 mV as a minimum requirement for pore formation^41^. By using a flow cytometric approach we have shown that salivaricin B is not able to induce pore formation or dissipate the cytoplasmic membrane potential of its targeted cells, exhibiting similar behavior to lacticin 481. This was accomplished with no evidence of short-lived pores or temporary dissipation of the membrane potential. While salivaricin B did not induce pore formation in this study, nisin did form pores. The pore formation caused by treatment with nisin did not have an immediate bactericidal effect towards *S. pyogenes* cells although the membrane permeabilization started shortly after exposure to nisin. The fluorescence intensity continued to increase during the exposure for 30 minutes. This is in agreement with other studies which showed that nisin may not have an immediate cell death action towards some bacterial strains contradicting with the conventional view of a rapid cell death after nisin addition^42^. Furthermore, membrane permeabilization usually occurs after target recognition and nisin/lipid II complex formation^43^. A previous study showed that the presence of pyrophosphate moiety in the target molecules is essential for target binding thereby suggesting that this moiety plays a vital role in the high activity and low cytotoxicity of nisin^44^. It is suggested that this phenomenon may slow down the death pattern caused by nisin in *S. pyogenes* since the degree of target recognition may differ from strain to strain. On the other hand, when used against *M. luteus* cells, nisin induced pore formation more rapidly and the slope also appeared steeper.

Salivaricin B, mutacin II, streptococcin SA-FF22 and lacticin 481 have one tryptophan residue at positions 17, 20, 18 and 19, respectively. Using spectrofluorometric analysis it was shown previously that adding artificial phospholipid vesicles to SA-FF22 in aqueous solution shifted the maximum fluorescence of the single tryptophan at position 18 from 352 nm to 337 nm (blue shift) indicating binding of SA-FF22 to the membrane vesicles^41^. However, no changes in the tryptophan fluorescence spectra of lacticin 481 were observed using the same approach, indicating that the tryptophan residue of lacticin 481 at position 19 plays no role in the interaction with cell wall precursor and vesicle membranes^12^. Previously, we have developed a peptide-membrane binding model using synthetic cationic peptides and bacterial vesicles to detect shift of the maximum fluorescence of the Trp residue^30^. In the current study, this same model has been used to track the change in the fluorescence of the tryptophan residue of salivaricin B at position 17. Our results show that salivaricin B does not penetrate bacterial membranes, as no typical blue shift in the tryptophan fluorescence was observed, even when different membrane vesicles isolated from *B. cereus* or *E. coli* were used. Salivaricin B is not active against intact *E. coli* cells. However, we have disrupted the cells by ultra-sonication and the resultant membrane fragments containing negatively charged phospholipids were tested in the tryptophan fluorescence experiment. We have chosen membrane vesicles from both Gram-Positive and Gram-Negative bacteria for use in our study. The aim of using *E. coli* membrane vesicles was to investigate the binding with minimum cell wall interference. Previous studies have shown that nisin A interacts with and causes perturbation of the cell membranes of spheroplasts prepared from *E. coli*^45^,^46^.

Salivaricin B was shown in this study to induce intracellular accumulation of the final soluble cell wall precursor UDP-MurNAc-Pentapeptide and ultimately inhibits cell wall biosynthesis. UDP-MurNAc-pentapeptide accumulation is usually induced by antibiotics inhibiting the late, membrane-bound steps of cell wall biosynthesis e.g. vancomycin^47^, bacitracin^48^ and ramoplanin^49^. Mersacidin and nukacin ISK-1 lantibiotics were also shown to inhibit the same cell wall precursor in *S. simulans* 22 cells^50^,^51^.

In this study, salivaricin B-treated *M. luteus* cells showed a significant reduction in cell wall thickness. This phenomenon was also previously observed in nukacin ISK-1-treated *B. cereus* cells and mersacidin-treated *S. simulans* cells^9^,^38^. Cell wall thinning can be attributed to interference with peptidoglycan biosynthesis resulting in failure to form adequate amounts of the final peptidoglycan chains. Additional and aberrant septum formation was evident in salivaricin B-treated cells and this deregulation and defection of the cell envelopes may lead to a failure to generate daughter cells. The bacterial cell wall is the osmotic barrier to the external environment and is a structural feature of cells, determining their shapes. The loss of shape in salivaricin B treated cells of both *S. pyogenes* and *M. luteus* strongly suggests that these cells lack functional cell walls. This mode of action is apparently irreversible, as salivaricin B activity was shown to be bactericidal against its targeted cells. Only partial lysis of *S. pyogenes* cells was observed after exposure to salivaricin B for 24 hours. Salivaricin B is the first member of the lacticin 481 lantibiotic group shown to cause aberrant septum formation in its targeted bacterial cells without also affecting membrane integrity. Salivaricin B has a specialised ABC transporter system (SboFEG) providing immunity and self-protection to the producer cells against the bactericidal action of the lantibiotic. This LanFEG system is very common in lantibiotics but cross immunity is extremely rare among lantibiotic producers^52^. Although Lipid II is an essential target for many lantibiotics, however, it was suggested previously that the lantibiotic resistance or sensitivity are independent of lipid II levels^53^. Moreover, the three-dimensional structure of a nisin resistance protein (SaNSR) has just been reported recently^54^.

It was reported recently that some non-lantibiotic-producing bacteria were shown to possess genes similar to the lantibiotic immunity systems. For example, genes encoding immunity homologues (*spiFEG*) have been found in *Streptococcus infantarius* subsp. *infantarius* BAA-102 with >50% homogeneity to that encoded in nisin U operon. Heterologous expression of these genes in lantibiotic sensitive *strain L. lactis subsp. cremoris* HP confers resistance to nisin U and other members of the nisin family^55^. Interestingly, the *sboFEG* immunity genes of salivaricin B were found in some *S. pyogenes* strains with 40–60% identity. Yet no salivaricin B resistant *S. pyogenes* strains have been reported previously. So far no significant resistance to salivaricin B was reported. Nevertheless, *Solobacterium moorei* CCUG39336 showed insignificant decrease in sensitivity to *S. salivarius K12* (producer of salivaricins A2 and B) when it was tested in antagonism assay over 10 repetitions^56^. Further work on salivaricin B immunity and resistance may provide vital insights into the emerging evolution of bacterial strains which develop new strategies to avoid elimination due to lantibiotics in the oral cavity.

## Materials and Methods

### Bacterial strains and culture conditions

The salivaricin B producer strain, *Streptococcus salivarius* K12, was grown on Mitis salivarius agar (MSA) or Columbia agar base supplemented with 5% whole human blood and 0.1% CaCO_3_ (BaCa). M17 medium supplemented with 2% yeast extract, 1% sucrose, 0.1% CaCO_3_ and 0.7 agarose (M17YESUCa) was used as the salivaricin B production medium.

Indicator strains *Micrococcus luteus* ATCC10240 and *Corynebacterium spp* GH17 were grown either on Mueller Hinton agar (MHA) or Trypticase Soy agar (TSA). *Streptococcus pyogenes* ATCC1234 and *Streptococcus mutans* GEJ11 were grown either on BaCa plates or TSA. For MIC tests, M17 medium supplemented with 1% glucose (GM17) was used to propagate the streptococcal strains. All streptococci were grown in a microaerophilic atmosphere using GasPak EZ CO2 Container System, BD at 37 °C. *M. luteus* and *Corynebacterium spp* were grown aerobically at 37 °C. *Lactococcus lactis* subsp. *cremoris* HP was propagated in GM17 at 30 °C. All media were purchased from Difco, BD. Nisin A was purchased from Sigma-Aldrich. All solvents and chemicals were purchased from Merck, Germany. Nisin A stock solution was prepared as described previously^57^.

### Deferred antagonism assay

*S. salivarius* K12 grown overnight on BaCa was used to inoculate 5 mL of PTNYSMES medium^24^ and incubated for 18 h at 37 °C using GasPak EZ CO_2_ Container System, BD. Two microliter of the resultant culture was spotted on BaCa plates and incubated under the same conditions recorded above. K12 cells were killed using chloroform vapors by inverting the plate over a filter paper soaked with chloroform for 30 minutes, followed by aeration of the plate for another 30 minutes to remove chloroform vapour residues. Indicator strains were grown either on BaCa (for *S. pyogenes* and *S. mutans*) or on MHA (for *M. luteus* and *Corynebacterium Spp*) and a few colonies were swabbed from the plates and suspended into PBS buffer (pH: 7) prior to diluting the resultant bacterial suspension to OD_600_ = 0.1. One mL of each of the diluted bacterial suspensions was added to 4 mL of Trypticase soy soft agar (0.5% bacteriological agar) and poured over the chloroform-killed K12 spot cultures. The plates were incubated for 18 h at 37 °C aerobically for both *M. luteus* and *Corynebacterium spp* and in CO_2_ Container System for *S. pyogenes* and *S. mutans* as mentioned above. Inhibition zones surrounding the producer strain were observed, indicating the inhibitory activity of the secreted lantibiotic. This method was repeated in triplicate and showed consistently reproducible results.

### Salivaricin B purification

*S. salivarius* K12 was grown for 18 hours in TSB at 37 °C in a microaerophilic atmosphere (GasPak EZ CO2 Container System, BD) before it was used to inoculate 150 M17YESUCa plates using sterile cotton swabs. The inoculated plates were incubated as mentioned above after which the whole cultures were scraped from the petri dishes, cut into small pieces and transferred into a 2000 mL beaker. This preparation was kept frozen at −40 °C for overnight before it was allowed to thaw at 50 °C in a water bath for 2 hours. The resultant extract was centrifuged at 10,000 rpm for 30 minutes and the supernatant was passed through Minisart 0.22 μm filter (Sartorius, Germany). The resultant liquid was passed through Amberlite XAD 16 column (Sigma, France) pre-equilibrated with methanol and water. The column was washed with 1 liter of distilled water followed by 500 mL of 50% methanol in water. Lantibiotic activity was eluted with 300 mL of 95% methanol adjusted to pH 2 using HCl. The methanol was evaporated using a rotary evaporator at 40 °C under reduced pressure and the resultant lantibiotic-containing preparation was tested using a spot-on-lawn technique. Briefly, two fold dilutions of the active fraction were tested by spotting 20 μl on MHA and the spots were left to dry before the indicator strain (18 hours old *M. luteus* ATCC10240 adjusted to OD_600_ = 0.1 using PBS pH 7.2) was applied as a lawn on the top of the agar plate using a sterile cotton swab. Arbitrary units (AU) per milliliter were defined as the reciprocal of the highest dilution factor that showed inhibition of the indicator strain. Salivaricin B was further purified by HPLC (Waters) on Chromolith SemiPrep RP-18e 100–10 mm column with gradient of 20–50% acetonitrile in water (v/v) over 60 minutes at a flow rate of one mL per minute. The run was monitored using UV detector at 214 nm. The fractions were collected manually every minute and the acetonitrile was evaporated using EYELA centrifugal evaporator CVE-2000 (Tokyo, Japan) equipped with vacuum pump. The fractions were tested as mentioned above. After identifying the retention time of salivaricin B elution, several runs were performed and a consistent and reproducible chromatogram was achieved at the defined retention time. To further analyze salivaricin B purity, active salivaricin B eluted from the previous HPLC run was subjected to Aeris PEPTIDE 3.6 u XB-C18 250x4.6mm column equilibrated with acetonitrile and water. Active fractions were subjected to 4800 *Plus* MALDI TOF/TOF Analyzer to determine the molecular weight.

### Minimal inhibitory concentration (MIC) and IC50 determination

MIC was determined using broth microdilution method. Two fold dilutions of salivaricin B or nisin A lantibiotics were prepared in adequate media in polypropylene 96-well plate (Nunc) where each well contained 50 μl of each lantibiotic preparation. Overnight cultures of each test bacteria were diluted to 7 × 10^5^ CFU.mL^−1^ using fresh medium and 50 μl of the diluted bacteria was added to every well. Wells with no bacteria added served as negative controls of no bacterial growth (blank). Wells containing bacterial culture only without any lantibiotic added served as positive growth control. The plates were incubated at a suitable temperature and under suitable conditions. After 18 hours of incubation, the highest lantibiotic dilution which inhibited 90% of the bacterial growth was considered as the MIC. The IC50 values were measured as mentioned previously^58^ and the calculation was carried out using The IC50 Tool Kit (http://ic50.tk/).

### Time killing assay

The mode of inhibitory activity of salivaricin B was determined by measuring the decrease with time in the CFU of targeted bacterial strains. Both *S. pyogenes* ATCC1234 and *M. luteus* ATCC10240 were used in this assay as sensitive strains. Ten hours old bacterial cultures were centrifuged at 2,000 rpm for 5 minutes to pellet the cells before washing with ice-cold 5 mM sodium phosphate buffer pH 7.2. Each strain was washed twice and resuspended in the same buffer to the original culture volume. The bacterial suspension was mixed at 1:1 ratio with either salivaricin B or nisin A lantibiotics at a concentrations of 10 x MIC and incubated at 37 °C. Bacterial suspensions mixed with 5 mM sodium phosphate buffer pH 7.2 were served as a control. Survivors were determined at intervals by plating serial dilutions of the test and control mixtures on TSA and incubated at 24 hours at 37 °C.

### Pore formation assay

To investigate the ability of salivaricin B to generate pores into the targeted bacterial membranes, SYTOX Green probe was used as mentioned previously^23^ with some modifications. *S. pyogenes* ATCC12344 was grown in GM17 under the same conditions mentioned above until mid-exponential phase was achieved (10^4^–10^5^ CFU.mL^−1^). The bacterial culture was combined with SYTOX Green (final concentration 5 μM) before ninety microliter of this suspension was transferred to MicroAmp Fast Optical 96-Well Reaction Plate (Applied Biosystems, Life Technologies, USA). The fluorescence was monitored for 10 minutes until stable base line was achieved. Ten microliter of pure salivaricin B (10X MIC) was added to this suspension and fluorescence signal from membrane-compromised bacteria labelled with SYTOX Green stain was detected with excitation and emission at 494 nm and 521 nm respectively using the Real-Time PCR as a fluorescence detection method as mentioned previously^59^. The same experiment was carried out using *M. luteus* ATCC10240 grown in MHB until OD_600_ = 0.5. SYTOX Green labeling and detection was carried out as mentioned above. Nisin was also tested as a known pore forming lantibiotic at 10 X MIC. Sodium phosphate buffer was added to another sample instead of lantibiotic and served as a negative control. All the samples were performed with three biological replicates.

### Estimation of membrane potential

Molecular dye 3,3-diethyloxacarbocyanine iodide DiOC2(3) was used to investigate the ability of salivaricin B to dissipate membrane potential of sensitive targeted strain. Cultures of *M. luteus* ATCC10240 were grown at 37 °C for 6 hours with orbital shaking at 100 rpm in MHB medium and then diluted to OD_600_ = 0.1 using fresh MHB. Diluted cultures were combined with DiOC2(3) at final concentration of 2 μM. Glucose (1 mM) and HEPES (1 mM) were added before the samples were incubated at room temperature for 30 minutes. Salivaricin B and nisin A were added at final concentrations of 10 X MIC and samples were further incubated for 20 minutes. The protonophore Carbonyl cyanide m-chlorophenyl hydrazone (CCCP) was also tested as a positive control. Distilled water was added instead of antibiotic to the negative control sample. Changes in cell-associated DiOC2(3) fluorescence were measured with BD FACSCanto II flow cytometer (excitation at 488 nm) using argon laser.

### Fluorescence measurements

Fluorescence spectra were recorded on a Shimadzu RF-5301 PC spectrofluorophotometer. In all the experiments, samples were measured in a 1 cm path-length quartz cell at 23 ± 1 °C. The concentration of all species was 0.05 mM in a phosphate buffer (10 mM) of pH 7.4. The reported values are the average of three measurements. Bacterial membrane vesicles were prepared from cultures of *Bacillus cereus* and *E. coli* as described previously^30^. The fluorescence experiments were performed under the same concentration of Salivaricin B. This was achieved by dividing a buffer solution that contains Salivaricin B into three portions. One portion was used as a reference, or a control, without any membrane. Membranes of *B. cereus* and *E. coli* were then each added to one of the other portions. In order to compensate for the amount added from each membrane, similar volume form the buffer was added to the control solution. We checked the buffer alone and there was no fluorescence in the spectral region (295–550 nm) which rules out any contribution from the buffer to the observed signal. Since *B. cereus* and *E. coli* membranes show fluorescence signal in the same spectral region, in two other sets of experiments, the control in each one was either membranes of *B. cereus* or *E. coli*.

### Intracellular accumulation of the peptidoglycan cell wall precursor (UDP-MurNAc-pentapeptide)

UDP-MurNAC-pentapeptide cytoplasmic pool was analyzed as described previously^60^,^61^,^62^ with some modifications. *M. luteus* ATCC10240 cells were grown overnight in TSB on orbital shaker at 150 rpm, 37 °C. The culture was diluted 1/100 (v/v) using fresh medium and further incubated under same conditions mentioned above until OD_600_ reached 0.7. The culture was supplemented with chloramphenicol (final concentration 130 μg/mL) and further incubated for 15 minutes. Then the culture was divided into three equal samples in three 50 mL sterile tubes. Vancomicin was added to the first tube at 10X MIC. The second tube was supplemented with salivaricin B (10X MIC) and the third tube was served as a control without antibiotic addition. The three tubes were further incubated for an hour before samples were centrifuged at 3,000 rpm for 30 minutes and the supernatant was discarded. The resultant cell pellets were extracted with boiled water for 20 minutes before centrifugation at 13,000 rpm for 15 minutes. The supernatant was freeze dried and the resultant powder was dissolved in 400 μl of 5 mM sodium phosphate buffer pH 5.2. Intracellular accumulation of UDP-MurNAc-pentapeptide was analyzed by RP-HPLC using 5 mM sodium phosphate buffer pH 5.2 as the mobile phase under isocratic conditions on Chromolith SemiPrep RP-18e 100–10 mm column. The run was monitored using UV detector at wavelength of 260 nm at a flow rate of 1 mL.min^−1^.

### Transmission Electron Microscopy (TEM)

Overnight cultures of both *M. luteus* ATCC10240 and *S. pyogenes* ATCC1234 grown in TSB were centrifuged at 3,000 rpm for 15 minutes. The cells were washed twice with ice-cold 10 mM phosphate buffer pH 7.2 before they were suspended into the same buffer to the original culture volume. The bacterial suspensions were incubated with either salivaricin B or nisin A at 10 x MICs for 30 or 120 minutes or for overnight. Control samples included bacterial suspension incubated with distilled water. The cells were fixed using 4% glutaraldehyde in 10 mM phosphate buffer pH 7.2 for overnight at 8 °C. After 3 washes in cacodylate buffer, the pellets were incubated for 2 hours in OsO_4_:cacodylate buffer (1:1) and then the samples were incubated with cacodylate buffer alone for overnight. After 3 washes in distilled water, the samples were washed with gradient concentrations of ethanol (35%, 50%, 70%, 95% and 3 times 100%) followed by two washes with propylene oxide, one wash with propylene oxide:epon mixture (1:1) and one wash with propylene oxide:epon mixture (1:3). After that the samples were incubated with epon for overnight and embedded at 37 °C for 5 hours followed by 60 °C for overnight. After ultrathin sections were achieved (0.1 μm), the samples were coated on copper grids, stained with uranyl acetate and subjected to LEO-Libra 120 TEM (Carl Zeiss, Oberkochen, Germany).

## Additional Information

**How to cite this article**: Barbour, A. *et al*. New insights into the mode of action of the lantibiotic salivaricin B. *Sci. Rep.* **6**, 31749; doi: 10.1038/srep31749 (2016).

## Supplementary Material

Supplementary Information

## Figures and Tables

**Table 1:** Minimal inhibitory concentrations (MIC) and IC50 of salivaricin B and nisin A.

Indicator strain	Salivaricin B (nM)		Nisin A (nM)	
	MIC	IC50	MIC	IC50
*Corynebacterium spp* GH17	269	140.557 ± 7.067	39	16.4117 ± 0.567
*Lactococcus lactis* subsp*. cremoris* HP	1080	407 ± 34	39	17.7 ± 0.9
*Lactococcus lactis* ATCC11454	2160	891.7 ± 29.69	2500	523.5 ± 152.6
*Micrococcus luteus* ATCC10240	540	269.548 ± 41.33	312.5	128.641 ± 19
*Staphylococcus aureus* RF122	ND^†^	ND^†^	617.5	240 ± 35
*Streptococcus constellatus* ATCC27823	1080	451.4 ± 85	154.3	66.8 ± 6.859
*Streptococcus equisimilis* ATCC12388	2160	1177 ± 223.4	154.3	74.25 ± 7
*Streptococcus mutans* GEJ11	ND†	ND†	2500	903.9 ± 96.49
*Streptococcus pneumonia* ATCC6301	2160	810.242 ± 45.26	154.3	57.9 ± 2.27
*Streptococcus pyogenes* ATCC12344	2160	1435.48 ± 326	617.5	219.146 ± 41.79
*Streptococcus pyogenes* ATCC12348	2160	1263.53 ± 596	617.5	232.152 ± 51.8
*Streptococcus salivarius* K12	ND†	ND†	78	34.658 ± 3.408
*Streptococcus salivarius* NU10	2160	972.01 ± 218.7	78	23.4701 ± 1.825
*Streptococcus salivarius* YU10	2160	912.285 ± 72.11	312.5	136.315 ± 8.895
*Streptococcus sanguinis* ATCC10556	4320	1590.27 ± 110.8	154.3	57.753 ± 5.71

^†^Not determined, strain resistant to 8640 nM of salivaricin B.
