# Supplementary material for: New insights into the mode of action of the lantibiotic salivaricin B
Source: Sci Rep. 2016 Aug 16;6:31749. doi: 10.1038/srep31749 (PMC4985645; doi:10.1038/srep31749)
Supplement: Supplementary Information [file srep31749-s1.doc]

**New insights into the mode of action of the lantibiotic salivaricin B**

Abdelahhad Barbour^1^, John Tagg^2^, Osama K. Abou-Zied^3^, Koshy Philip^1^*

^1^Division of Microbiology, Institute of Biological Sciences, Faculty of Science, University of Malaya, Kuala lumpur, Malaysia.

^2^Department of Microbiology and Immunology, University of Otago, P.O. Box 56, Dunedin 9054, New Zealand

^3^ Department of Chemistry, Faculty of Science, Sultan Qaboos University, P.O. Box 36, Postal Code 123, Muscat, Sultanate of Oman.

* Corresponding author: kphil@um.edu.my

Key words: Lantibiotics, Salivaricin B, Mode of action.


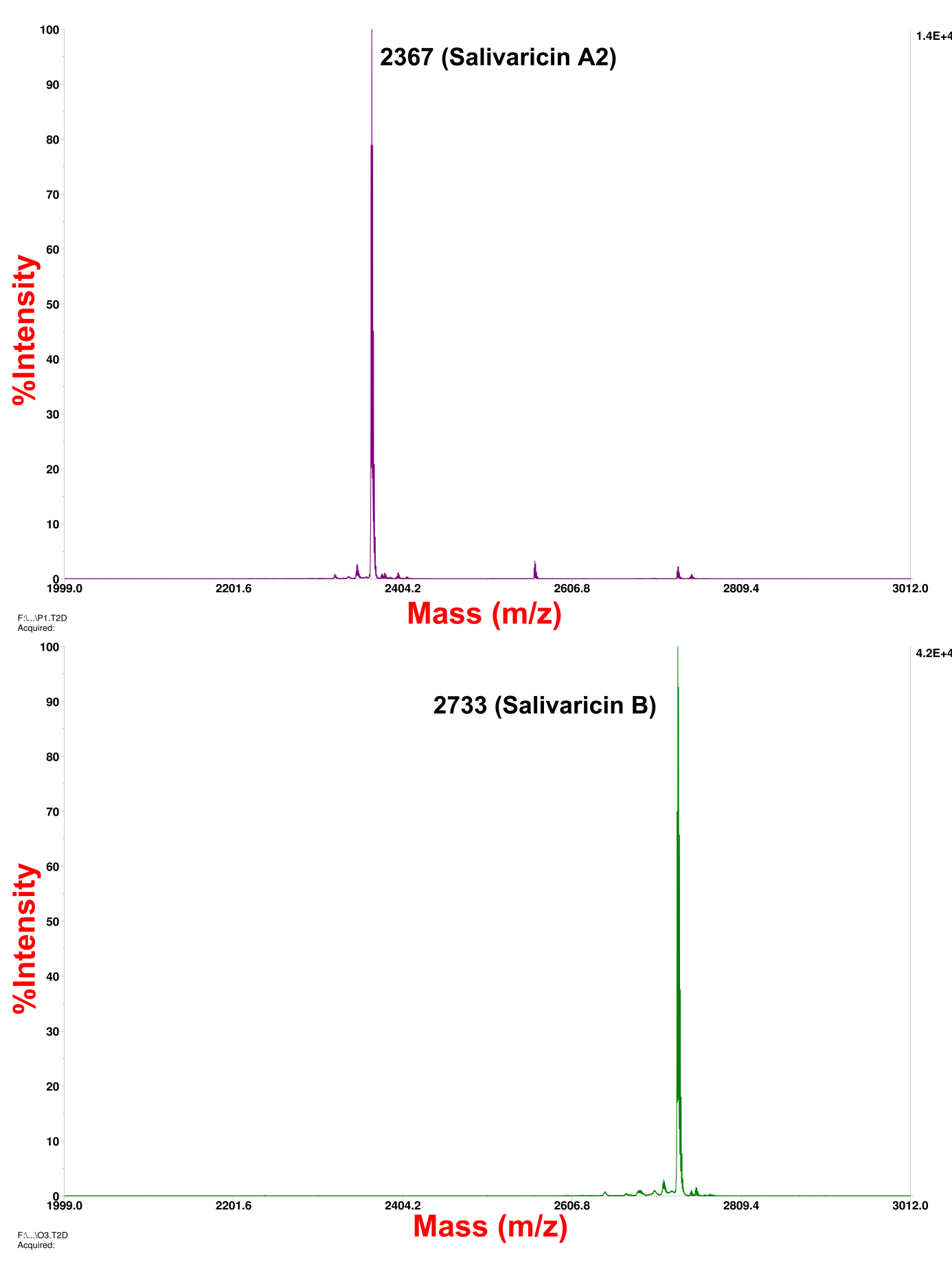


Figure S1: Single peak resolution MALDI-TOF (MS) analysis of salivaricin A2 and salivaricin B.


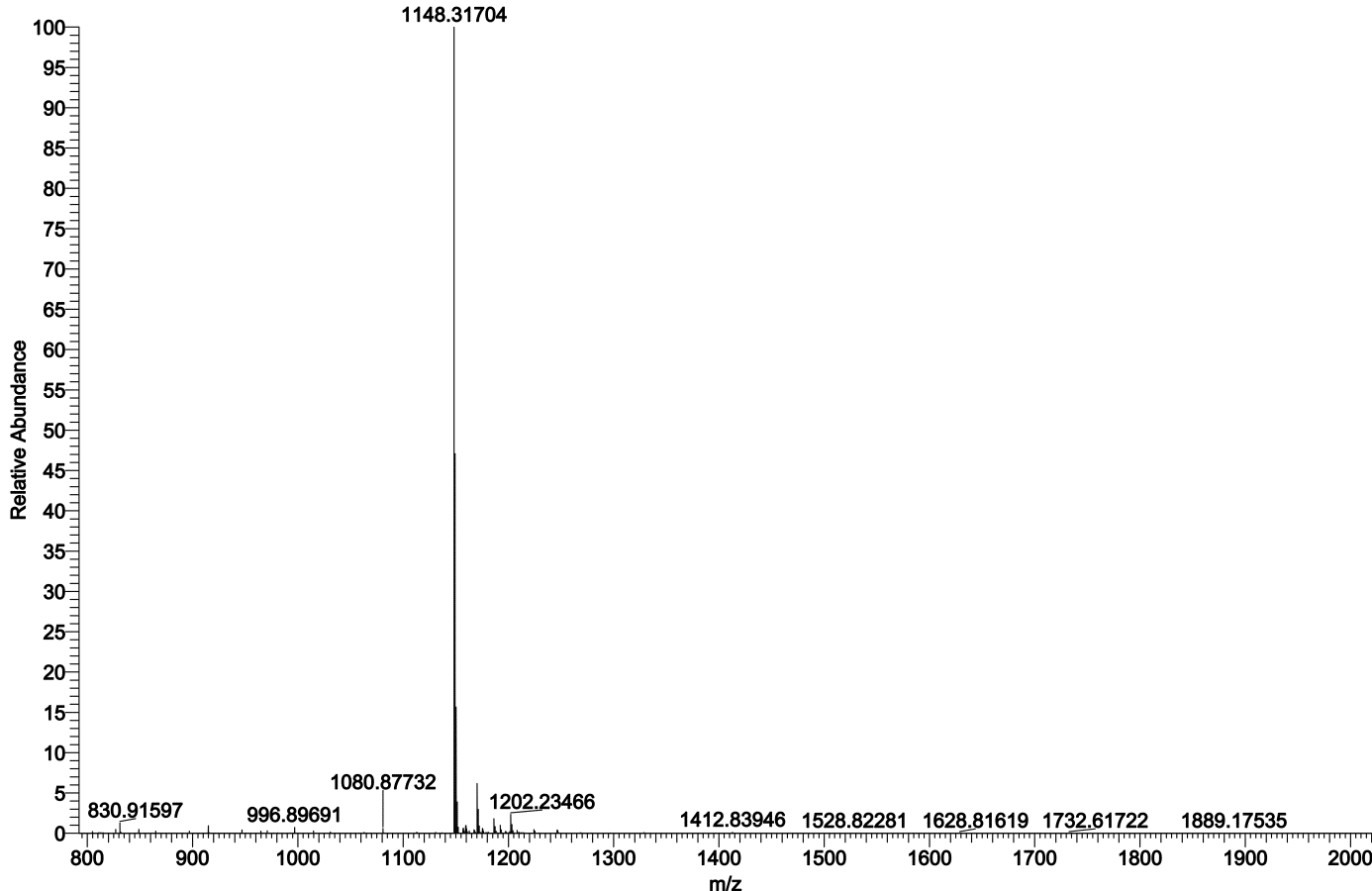


Figure S2: Identification of the accumulated cell wall precursor UDP-MurNAc-pentapeptide using Mass spectrometry analysis.
